# One Pot Synthesis of Nanofiber-Coated Magnetic Composites as Magnetic Dispersive Solid-Phase Extraction Adsorbents for Rapid Determination of Tetracyclines in Aquatic Food Products

**DOI:** 10.3390/molecules28217421

**Published:** 2023-11-03

**Authors:** Peipei Li, Junlu Bai, Pengfei He, Junjie Zeng

**Affiliations:** 1 Zhejiang Marine Fisheries Research Institute, Tiyu Road 28, Zhoushan 316021, China; 2 College of Food and Pharmacy, Zhejiang Ocean University, 1 South Haida Road, Zhoushan 316000, China

**Keywords:** C-nanofiber-coated magnetic composites, magnetic dispersive solid-phase extraction, tetracyclines, mass spectrometry, aquatic products

## Abstract

A magnetic adsorbent based on a C-nanofiber (Fe_3_O_4_@C–NFs) nanocomposite was synthesized using a simple one-pot co-precipitation method. The characterized results showed that the obtained C–nanofiber–coated magnetic nanoparticles had many attractive features such as a large specific surface area and a highly interwoven and branched mesoporous structure, as well as distinguished magnetism. The nanocomposite was then used as an adsorbent in the magnetic solid phase extraction (MSPE) of four typical tetracyclines (oxytetracycline, tetracycline, chlortetracycline, and doxycycline) in aquatic products. The TCs in the extract were determined using ultra-high-performance liquid chromatography–tandem mass spectrometry (UHPLC-MS/MS). Experimental variables of MSPE, including the sorbent amount, pH condition, adsorption and desorption time, and desorption solvent, were investigated and optimized systematically. The method validation indicated that the developed method showed good linearity (R^2^ > 0.995) in the range of 1.0–200 ng/mL. The average recoveries at the spiked levels ranged from 90.7% to 102.7% with intra-day and inter-day relative standard deviations (RSDs, *n* = 6) ranging from 3.72% to 8.17% and 4.20% to 9.69%, respectively. The limit of detection (LOD) and limit of quantification (LOQ) for the four kinds of TCs were 0.7 μg/kg and 2.0 μg/kg, respectively. Finally, MSPE based on C-nanofiber-coated magnetic nanoparticles was successfully applied to TC analysis in real aquatic products (grass carp, large yellow croaker, snakehead, mandarin fish, Penaeus vannamei, swimming crab, etc.). Compared with traditional extraction methods, the proposed method for TC analysis in aquatic products is more sensitive, effective, recyclable, and environmentally friendly.

## 1. Introduction

As a typical representative of hydrophilic antibiotics, tetracyclines (TCs) are widely used in animal husbandry and aquaculture for their broad antibacterial spectrum, good water solubility, and low cost [1,2] and play an important role in the prevention and treatment of bacterial infections, as well as growth promotion [3]. Toxicology studies of TCs indicate that these antibiotics show quite serious side effects, such as abdominal discomfort, epigastric pain, nausea, vomiting and anorexia, discoloration of teeth, and inhibition of bone growth in children. Furthermore, when entering the environment, TCs may lead to the induction and spread of drug-resistant bacteria, which poses a long-term potential threat to human health and the ecological environment [4,5,6,7,8]. Consequently, it is necessary to develop a fast, sensitive, and reliable method to detect and quantify TC residues in food samples.

TCs are usually detected in food using liquid chromatography coupled with UV [9,10], fluorescence [11,12], and mass spectrometry (MS) [13,14,15]. Among them, ultra-high-performance liquid chromatography–tandem mass spectrometry (UHPLC-MS) is more advantageous for its shorter analysis time, higher sensitivity, and excellent separation efficiency in quantitative analysis. Sample pretreatment is also a crucial step for accurate determination, considering the trace levels of target analytes and high levels of matrix interference. In recent years, magnetic solid phase extraction (MSPE) has been accorded considerable attention as a promising extraction approach. MSPE has the ability to adsorb and desorb analytes on a magnetic adsorbent with the help of an external magnetic field and could avoid the problems of column packing arising in conventional SPE [16,17]. MSPE can effectively shorten the pretreatment time and reduce the dosage of organic solvents, and it has the remarkable characteristics of recyclability and the advantages of being green, efficient, fast, and easy to operate. At present, MSPE has been successfully applied in the pretreatment of various antibiotics such as sulfonamides and quinolones in food matrices [18,19,20]. As adsorbents of MSPE, magnetic functional materials are the core of MSPE. The synthesis of highly efficient and specific adsorbents is a critical factor for the effectiveness of MSPE. Various functionalized magnetic materials have been fabricated and used as appropriate sorbents in MSPE for TC extraction in many kinds of matrices, such as Fe_3_O_4_@SiO_2_@FeO magnetic nanocomposite [21], iron–tannic nanoparticles [7], Fe_3_O_4_@[Cu_3_(btc)_2_] [22], magnetic graphene oxide/ZnO nanocomposite [23], and Fe_3_O_4_@graphene [21]. However, the current research on TC determination when using MSPE has certain limitations: firstly, most of the synthesis processes are complicated and the synthesis conditions are harsh and use expensive, toxic materials and solvents, resulting in certain environmental pollution. Secondly, MSPE is mainly used to extract or enrich TCs from water or milk [21,24,25,26] and is rarely applied to aquatic products. The complex substrate of aquatic products makes the successful application of MSPE more challenging.

With the deepening of the concept of green and sustainable chemistry, reducing the introduction of organic solvents and secondary pollution in material synthesis processes has become an important aspect of green separation chemistry. Therefore, low-cost, simple, and green material synthesis strategies are becoming the development mainstream. The realization of the accurate sensitive analysis of contaminants using a single material offers significant advantages in terms of cost, time, and ease of use. Among many magnetic materials, carbon-based nanomaterials, such as carbon nanofibers (C–NFs) consisting of graphene layers wrapped into cylinders and characterized by a high surface area, high adsorption capacity, and inertness in working solution environments, are more preferred as MSPE adsorbents in comparison with other carbonaceous nanomaterials. Some researchers developed an MSPE methodology based on C–NFs for the determination of tetracycline antibiotic residues in milk samples [27]. The adsorption of TCs onto the Fe_3_O_4_@C–NFs could contribute to π–π stacking, hydrogen bonding, electrostatic, and van der Waals forces between them. However, the application of carbon nanofibers for trace extraction of tetracycline from aquatic products has not been reported.

In the current study, new C–nanofiber-coated magnetic nanoparticles were synthesized using a simple one-step hydrothermal method, followed by structural characterization and adsorption performance evaluation. The developed nanocomposite adsorbent was applied in MSPE for four typical tetracyclines (oxytetracycline, tetracycline, chlortetracycline, and doxycycline) at trace levels in aquatic samples, which were to be determined using UHPLC-MS/MS. All the key factors affecting the efficiency of MSPE were systematically optimized. As discussed above, there has been no article reporting the use of the C–nanofiber-coated magnetic–MSPE method for the determination of TC residues in aquatic products so far. A magnetic adsorbent with low-cost and good adsorption properties was synthesized using a simple green one-step preparation method. This experiment can expand the application of carbon nanofibers in animal-source solid food such as aquatic products and solve the problem of slow column passage in the routine pretreatment of tetracycline drugs.

## 2. Results

### 2.1. Optimization of Ultra-Performance Liquid Chromatography–Tandem Mass Spectrometry Conditions

Based on the chemical structures and properties of TCs, a multiple reaction monitoring (MRM) mode and electrospray positive ion scanning (ESI^+^) mode were chosen for the MS method development. Acquisition parameters were optimized in ion spray mode by injecting direct infusion of individual standard solutions (1.0 μg mL^−1^) of TCs at a flow rate of 10 μL min^−1^. Protonated molecular ions [M+H]^+^ were chosen as precursor ions, and product ions were obtained by adjusting the cone voltage and collision energy from the precursor ions. The two ions with strong abundance and the least interference were selected as quantitative ions and qualifier ions, respectively. During analyte infusions, all related mass spectrometry parameters including capillary voltage, cone voltage, source temperature, desolvation gas temperature, and collision energy of the two most abundant transitions were optimized to achieve maximum sensitivity. The detailed optimized parameters and MRM transitions are presented in Table 1. The main fragment ions that may be generated by the precursor ions of TCs are [M+H-NH_3_]^+^, [M+H-H_2_O]^+^, and [M+H-NH_3_-H_2_O]^+^. By optimizing the impact energy, [M+H-NH_3_-H_2_O]^+^ fragment ions were found to be more abundant in the fragments from parent molecular ions of OTC, TC, and CTC; in other words, the daughter ions with a molecular mass of 35 lost after cracking had high corresponding strength and could be used as quantitative ions. For example, the parent ions of TC, OTC, and CTC are 445.1, 461.1, and 479.1, respectively, and the quantitative ions are 410, 426, and 443.9, respectively.

The use of UHPLC for methodology development provides superior chromatographic resolution, higher sensitivity, and shorter run times as compared with conventional liquid chromatography [12]. The BEH C_18_ column with a 1.7 μm particle size gave the best separation and a high analysis speed for the analytes. All four TCs were separated within 5 min, and the total run time obtained was just 7.0 min, which included 2.0 min for column stabilization. A 0.1% formic acid aqueous solution can achieve both enhancement of positive ion ionization intensity in MS and the best separation in UHPLC. The optimized gradient elution conditions are indicated in Section 2.3.

### 2.2. Preparation and Characterization of the Carbon Nanofiber Magnetic Material Composites

**Figure 1:**
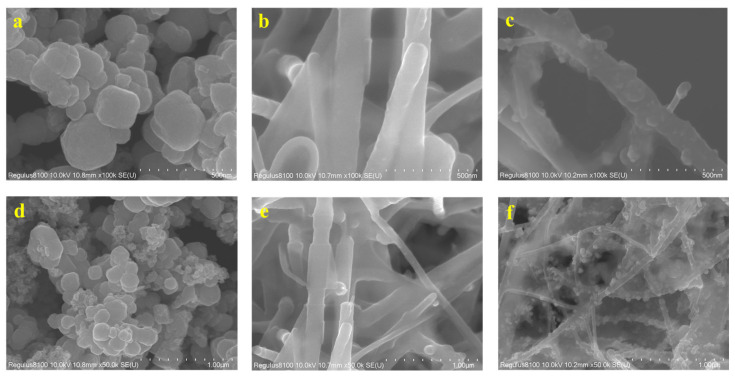
SEM images of Fe_3_O_4_ (**a**,**d**), C–NF (**b**,**e**), and Fe_3_O_4_@C–NF nanocomposites (**c**,**f**).

**Figure 2:**
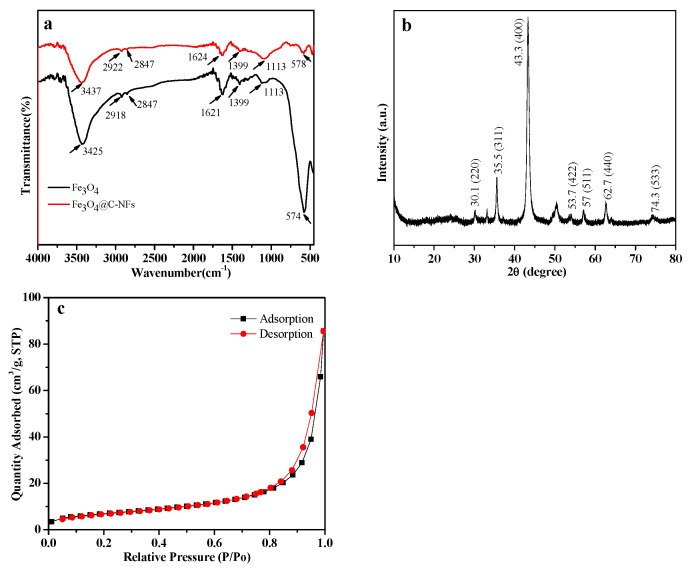
(**a**) FT–IR spectra of Fe_3_O_4_ and Fe_3_O_4_@C–NFs; (**b**) XRD spectrum of Fe_3_O_4_@C–NFs; and (**c**) nitrogen adsorption/desorption isotherms of Fe_3_O_4_@C–NFs.

Several characterized methods were performed to verify the Fe_3_O_4_@C–NF composites. A scanning electron microscope (SEM) was used to study the morphology characters of the Fe_3_O_4_ (Figure 1a,d), C–NFs (Figure 1b,e), and Fe_3_O_4_@C–NFs composites (Figure 1c,f). As depicted in Figure 1, the Fe_3_O_4_@C−NF composites possessed a large surface area and numerous sorption sites, and the Fe_3_O_4_ particles were homogeneously distributed on the fiber rods of the C–nanofiber. FT–IR spectra (Figure 2a) were used to verify the structure and functional groups of the Fe_3_O_4_@C–NF composites. The characteristic peaks at 3437 cm^−1^, 1624 cm^−1^, and 578 cm^−1^ were ascribed to O–H, H–O–H, and Fe–O stretching vibration, respectively. The spectrum of the Fe_3_O_4_@C–NF nanocomposite was consistent with Fe_3_O_4_ particle spectra since no peak shifts were observed. However, the peaks at 3425 cm^−1^ and 2918 cm^−1^ for Fe_3_O_4_@C–NFs shifted to 3437 cm^−1^ and 2922 cm^−1^, respectively, in the nanocomposite. The slight shift in peaks could be ascribed to the introduction of C–NFs into Fe_3_O_4_ particles. Therefore, the results of the FTIR analysis suggested that the Fe_3_O_4_@C–NF nanocomposite was successfully synthesized. X-ray diffraction (XRD) measurements were made to examine the crystal structure of Fe_3_O_4_@C–NFs. As shown in Figure 2b, a group of diffraction peaks at around 30.2° (220), 35.5° (311), 53.7° (422), 57.0° (511), 62.7° (440), and 74.3° (533) were related to Fe_3_O_4_ microspheres in the composites, and the observed diffraction peaks at 2θ: 43.3 (400) was for specific peaks of C–NFs. N_2_ adsorption–desorption isotherms were performed to obtain the specific surface area and pore size of Fe_3_O_4_@C–NFs. The BJH adsorption average pore width (4 V/A) of 10.7 nm was calculated using the Barrett–Joyner–Halenda (BJH) method, which could also be reflected in the pore size distribution curve (Figure 2c, inset). The Brunauer–Emmett–Teller (BET) surface area and total pore volume of the composites were 30.7 cm^2^ g^−1^ and 0.30 cm^3^ g^−1^, respectively (Figure 2c). The results give confirmation of a large specific surface area and a uniform pore radius distribution of the Fe_3_O_4_@C–NF composites.

### 2.3. Optimization of MSPE with the Nanocomposite Adsorbent

In order to obtain optimum MSPE conditions for rapid isolation and extraction of TCs with low solvent consumption in aquatic products, several parameters affecting MSPE including extraction solvent, adsorbent amount, adsorption and desorption time, pH, type, and volume of eluent were systematically investigated. Extraction recoveries were calculated for the evaluation of extraction efficiency.

#### 2.3.1. Effect of pH of the Sample Solution and Type of Extraction Solvent

**Figure 3:**
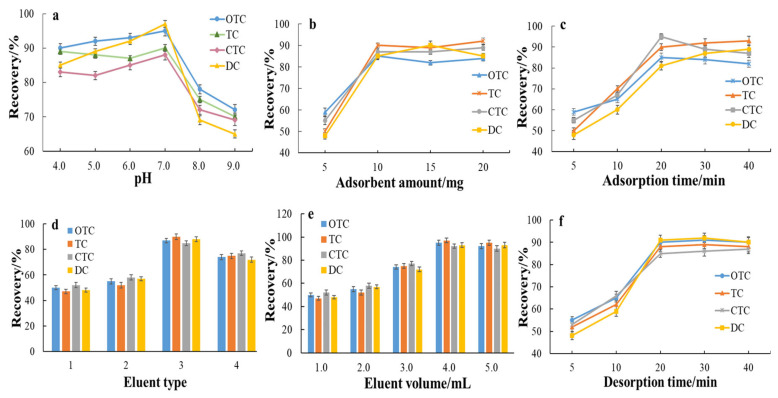
Effect of pH (**a**), adsorbent (**b**), adsorption time (**c**), eluent type (**d**), eluent volume (**e**), and desorption time (**f**) on the recovery response of the Fe_3_O_4_@C–NF nanoparticles in MSPE application to the 4 kinds of TCs.

In this experiment, due to the complex matrix of aquatic products, purified water was first used as a model solution, and the effect of pH on the adsorption efficiency was studied by adding TCs to the model solution. Different pH values (4.0, 5.0, 6.0, 7.0, 8.0, and 9.0) of water were studied, and the other MSPE conditions were fixed. As shown in Figure 3a, favorable adsorption efficiencies were obtained within a range of pH 4–7 and progressively decreased when the pH further increased to 8.0. When the pH ranged from 8 to 9, the adsorption efficiency continued to decrease. In this experiment, the sample extract was concentrated with a gentle nitrogen stream and then redissolved with deionized water to obtain a sample solution. The acidity of deionized water is approximately 6.5~7.0 in general; hence, the MSPE procedure was used directly to extract TCs from the sample solution without pH adjustment.

#### 2.3.2. Effect of the Type of Extraction Solvent

Different from water or liquid samples, the selection of an appropriate extraction solvent when applying the MSPE method to aquatic products is important and critical. The suitable extraction solution should not only achieve high extraction efficiency but also ensure high adsorption of the magnetic material to the target analytes. Containing hydroxyl groups in structures, TCs show high stability and good solubility in acidic aqueous solutions. The weakly acidic Na_2_EDTA–Mcllvaine buffer solution was usually used for the extraction of TCs from aquatic products. According to the pretreatment method of QuEChERS, an acidified acetonitrile aqueous solution and an appropriate amount of Na_2_EDTA were used to extract the analytes, and a high extraction rate was obtained. In this work, the adsorption of magnetic materials on TCs in pure water, a Na_2_EDTA–Mcllvaine buffer solution with different pH values, and a glacial acetic acid–acetonitrile–water mixed solution (1:84:15, *v*/*v*) without adding real samples was tested first. The results showed that the adsorption rate could reach more than 90% in both pure water and the glacial acetic acid–acetonitrile–water mixed solution but only about 50% in the Na_2_EDTA–Mcllvaine buffer solution, which was possibly due to the inhibition of adsorption by complex ions in citric acid. Hence, the glacial acetic acid–acetonitrile–water mixed solution (1:84:15, *v*/*v*) was used to extract TCs from fortified real samples, followed by adsorption with magnetic material. The results showed that the adsorption rate was approximately zero. The results showed that the adsorption process could not be carried out when the adsorbent material was directly placed in the extraction solution. It is necessary to convert the solution to high-proportion water with dilution or redissolve after concentration before adsorption.

#### 2.3.3. Effect of Adsorbent Amount

The adsorbent amount is an important parameter that affects the adsorption of target analytes. Given that TCs can be detected at trace level with the help of the high sensitivity of MS, and that the synthesized adsorbent has good specific surface area and adsorption properties as mentioned above, the amount of adsorbent does not need to be too large. The optimization of Fe_3_O_4_@C–NFs usage was performed by varying its amount from 5 to 20 mg (5, 10, 15, and 20 mg). As shown in Figure 3b, the extraction recoveries of TCs increased as the adsorbent weight increased from 5.0 mg to 10.0 mg and had no remarkable improvement since more than 20 mg of the adsorbent was added. The result demonstrated that 10.0 mg of adsorbent could achieve excellent extraction performance for TCs in the given conditions.

#### 2.3.4. Effect of Adsorption Time

In MSPE, adsorption time is one of the decisive factors that greatly influences extraction efficiency. Efficient contact of the adsorbent with the sample solution containing the analytes is an important criterion for obtaining high extraction efficiency. Different adsorption times ranging from 5 to 40 min were investigated. As seen in Figure 3c, the extraction efficiency gradually increased as the time extended from 5 to 20 min, and there appeared to be no significant change as the time extended beyond 20 min, indicating that the rapid distribution equilibrium between TCs and adsorbent was established within 20 min. Therefore, the adsorption time was set to 20 min.

#### 2.3.5. Effect of Eluent Type

One of the most critical steps of MSPE is the thorough elution of the analyte adsorbed on the magnetic material. A proper type of eluent should first be able to elute the analyte from the adsorbent exhaustively to obtain reliable and reproducible analytical results, and second, it should be able to dissolve a high percentage of analyte and be suitable for subsequent mass spectrometry analysis. Firstly, different kinds of commonly used eluents including acetonitrile, methanol, methanol/water (1:1, *v*/*v*), and formic acid in methanol (2%, 5%, 10%, *v*/*v*) were tested. The results indicated that TCs cannot be leached out from the magnetic sorbent by 100% MeOH and ACN, and the elution efficiency ranged from 2.7% to 7.4% for four TCs with formic acid in methanol (2%, 5%, 10%, *v*/*v*). According to some studies [21,28], a certain concentration of oxalic acid was beneficial to the disruption of the coordination interaction between adsorbent and TCs, which were based on the protonation of the carboxylic acid of the antibiotic. Therefore, several eluents consisting of different proportions of acetonitrile, methanol, and oxalic acid were evaluated to desorb TCs from the magnetic microspheres. The results are shown in Figure 3d. The experimental results indicated that TCs can be effectively released by desorption with the ACN-0.02 mol L^−1^ oxalic acid solution (1:8, *v*/*v*).

#### 2.3.6. Effect of Eluent Volume and Desorption Time

The effect of eluent volume (1.0–5.0 mL) on the recoveries was also investigated. Satisfactory recoveries were obtained when the eluent volume was 4.0 mL (Figure 3e). In order to study the influence of desorption time, 5, 10, 20, 30, and 40 min were investigated, and the other SPE conditions were fixed. It is clearly demonstrated in Figure 3f that the extraction efficiency increased as the time extended from 5 to 20 min, while prolonging the time to 30 min showed no significant increase. Taking the extraction amount and the extraction time both into consideration, 20 min of the vortex treatment is enough to elute TCs.

### 2.4. Method Validation

**Figure 4:**
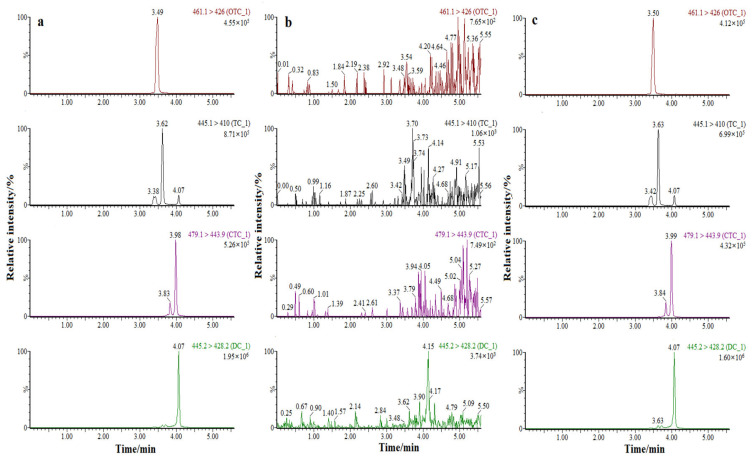
MRM chromatogram (MS/MS) of the 4 kinds of TCs at a concentration of 5.0 μg kg^−1^ ((**a**), a standard solution of 2.0 ng/mL; (**b**), a blank fish sample; and (**c**), a spiked fish sample containing TCs at a concentration of 2.0 ng/mL).

The validation results are listed in Table 2 and Table 3. Favorable linearities for four TCs were obtained in the range of 1.0–200 ng/mL (1.0, 2.0, 5.0, 10.0, 25.0, 50.0, 100.0, and 200.0) with correlation coefficients (r^2^) > 0.995. In addition, individual residual deviation was below 20% for each one of the calibration standards. The back-calculated concentrations of the calibration standards were within ±15% of the nominal values. Acceptable recoveries were in the range of 80.7% to 97.7%. Precision was examined using intra- and inter-day RSDs, which were found to be in the range of 3.72% to 8.17% (*n* = 6) and 4.20% to 9.69% (*n* = 6), respectively. Based on S/N = 3 and S/N = 10, the LOD values and the LOQ values were 0.7 μg/kg and 2.0 μg/kg, respectively. For specificity, no interference of endogenous compounds or ion traces was found in the matrices studied, as seen in Figure 4a–c. Peaks that could be assigned to endogenous compounds did not interfere with that of the analyte, and no interfering peaks from endogenous compounds were found in the retention time of the target analyte. Matrix effects were 97.7–103.7%, which is well within acceptable ranges (<20%).

### 2.5. Reproducibility and Recyclability of Fe_3_O_4_@C–NFs

For reproducibility, the Fe_3_O_4_@C–NF composites that were prepared in different batches were used for the MSPE procedure, according to Section 3.5, and the inter-batch precisions were calculated by subtracting the method precision from relative standard deviations (RSDs) of the measured content of the analytes. The obtained inter-batch precisions were in the range of 5.7–6.3% (Table 2), implying that the Fe_3_O_4_@C–NF composites were prepared with good reproducibility.

The reusability of the adsorbent is an outstanding advantage and critical aspect of MSPE from economic and environmental points of view. The reusability experiment was performed by washing the adsorbent with sufficient amounts of deionized water and methanol after use and reusing it for the next MSPE procedure, according to Section 2.5. There was no significant difference in the extraction efficiency between newly synthesized and recycled composites. Based on the obtained results, the adsorbent can be reused at least 10 times with <5% loss in the extraction recoveries.

### 2.6. Comparison of MSPE with Other Methods

To confirm the MSPE performance of the Fe_3_O_4_@C–NFs, reported studies regarding the determination of TCs using different pretreatment methods in different matrices were compared (Table 4). As shown in Table 4, the proposed method exhibited several advantages. First, the sensitivity of the proposed method was significantly higher than other HPLC methods and was comparable to LC/MS methods that use SPE. More critically, in contrast to conventional SPE methods, the proposed method did not require complicated steps, including activation, leaching, or elution, and has the advantages of convenience and recyclability. Second, less sorbent was required to efficiently concentrate trace TCs due to the large specific surface area and high sorption. Furthermore, it can also be seen from Table 4 that at present, magnetic materials used as adsorbents for MSPE detection of tetracycline drugs are mainly used in environmental water bodies, milk, and other liquid substrates, and few have been successfully applied in solid substrates such as aquatic products. The proposed method has expanded the application range of magnetic materials. Compared with other adsorbents, there are no toxic or harmful reagents used in the synthesis procedure of Fe_3_O_4_@C–NFs, and the synthesis method is simple and environmentally friendly.

### 2.7. Analysis of Real Samples

The established method was further applied to the detection of TCs in aquatic product samples. Samples from different kinds of aquatic products (grass carp, large yellow croaker, black porgy, snakehead, mandarin fish, Penaeus vannamei, Parapenaeopsis hardwickii, red shrimp, and swimming crab), which possibly contain TCs, were collected from several markets and aquaculture farms in Zhejiang Province. The four kinds of TCs were not detected in the above aquatic products. The spiked recoveries ranged from 81.5 to 103.7%. Nevertheless, it is still necessary to strengthen the residual monitoring of TCs in aquatic products. The MRM chromatograms of the real samples are shown in Figure 4.

## 3. Materials and Methods

### 3.1. Chemicals and Reagents

C–nanofibers at 50–200 nm in diameter and 1–15 μm in length were purchased from Jiangsu Xianfeng nanomaterials Technology Co., Ltd. (Nanjing, China). Ferric trichloride hexahydrate (FeCl_3_⋅6H_2_O), sodium acetate (CH_3_COONa), ethylene glycol, and oxalic acid dehydrate were all analytical grade and purchased from Sinopharm Chemical Reagent (Shanghai, China). Methanol (MeOH), acetonitrile (ACN), acetic acid, and formic acid were HPLC grade (99.9%) and obtained from Merck (Darmstadt, Germany). Deionized water used throughout the experiment was prepared using a Milli-Q system (Millipore, Bedford, MA, USA).

Tetracycline standards including oxytetracycline (OTC), tetracycline (TC), chlortetracycline (CTC), and doxycycline (DC) were hydrochloride salts and purchased from Sigma-Aldrich (St. Louis, MO, USA).

Individual standard stock solutions of OTC, TC, CTC, and DC were converted from hydrochloride salt to free base and prepared by dissolving each compound in methanol to reach a concentration of 100 μg/mL. Stock solutions were stored at 4 °C in the dark and were proven to be stable under storage conditions. Working mixed standard solutions were prepared by diluting the stock solutions with deionized water before use.

### 3.2. Apparatus

The morphological evaluation of synthesized magnetic C-nanofibers was carried out using a scanning electron microscope (SEM) (Hitachi Regulus8100, Tokyo, Japan). The functional groups of the sorbent were confirmed using an FT-IR spectrometer (Thermo Scientific Nicolet iS20, Waltham, MA, USA). The Brunauer–Emmett–Teller (BET) surface area, nitrogen sorption isotherms, and pore size distribution were measured at 200 °C using an ASAP 2460 Surface Area and Porosity Analyzer (Micromeritics Instrument Corporation, Norcross, GA, USA). Powder X-ray diffraction (XRD) measurements were performed with a PANalytical X’Pert’3 Powder X-ray diffractometer (PANalytical BV, Almelo, The Netherlands) using Cu K α radiation (λ = 0.15406 nm) in the 2θ range of 10–80° at room temperature. Vortex mixing was performed using an IKA Vortex Genius 3 vortex mixer (IKA, Staufen, Germany). For all the chemicals and reagents, an AB204-N analytical balance (Mettler Toledo, Greifensee, Switzerland) was used for weighing. Vibrationing was performed using a thermostat gas bath vibrator (Changzhou Guowang Instrument manufacturing Co., Ltd., Guangzhou, China). Balanced oscillator stirring was performed using an HD2004W constant speed mechanical stirrer (Sile, Shanghai, China). All pH measurements were carried out using H1 9811-5 (HANNA Instrument, Smithfield, RI, USA).

### 3.3. LC-MS/MS Conditions

Chromatography was performed using a Waters Acquity UPLC^TM^ system (Waters, Milford, MA, USA) coupled with Quattro Premier XE Micromass triple–quadrupole mass spectrometer (Waters, Manchester, UK). Chromatographic separation was achieved on a Waters BEH C_18_ chromatography column (2.1 × 100 mm, 1.7 μm particle size) and performed using a binary gradient mobile phase consisting of a 0.1% formic acid solution (A) and acetonitrile (B) at a flow rate of 0.3 mL min^−1^. The gradient was designed as follows: 0~1.0 min, 90% A; 1.0~2.0 min, 90–70% A; 2.0~3.5 min, 70–40% A; 3.5~5.0 min, 40% A; 5.0~5.5 min, 40–90% A; and 5.5~7.0 min, 90% A. The temperature of the column and the autosampler was set at 35 °C and 10 °C, respectively. The injection volume was 10 μL including a needle wash function. MassLynx software 4.1 was used for instrument control and efficient data acquisition.

The mass spectrometer (MS) was operated with electrospray ionization (ESI) in positive ion mode. Mass spectrometric analyses were performed in the multiple reaction monitoring (MRM) mode. The optimized MS/MS parameters were as follows: cone and desolvation gas: nitrogen (99.9% purity), collision gas: argon (99.9999% purity), source temperature: 150 °C, desolvation temperature: 600 °C, capillary voltage: 3.5 kV, cone gas flow: 50 L h^−1^, and desolvation gas flow: 600 L h^−1^.

### 3.4. Synthesis of Fe_3_O_4_@C–NF Composites

**Figure 5:**
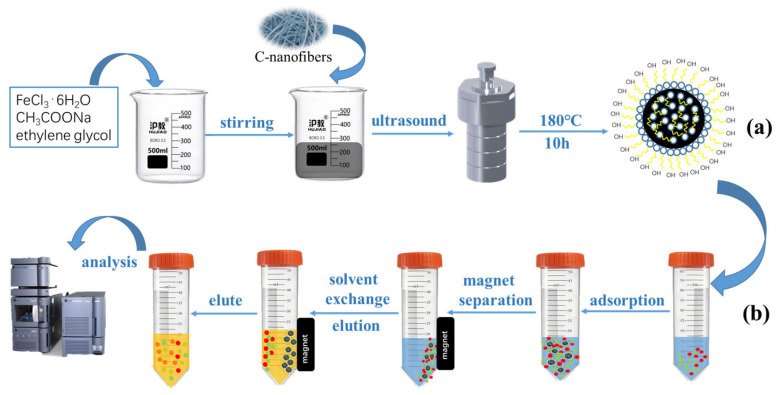
A schematic diagram for the preparation of the Fe_3_O_4_@C–NF nanocomposites (**a**) and their application for TCs extraction (**b**).

C-nanofiber-coated magnetic nanoparticles were synthesized using the hydrothermal synthesis method according to a previous report [27], with some modifications. The synthetic protocol is shown in Figure 5a. Briefly, 2.5 g FeCl_3_⋅6H_2_O and 4.0 g CH_3_COONa were dispersed in a solution containing 25 mL deionized water and 50 mL ethylene glycol by stirring. Afterward, 0.50 g of C-nanofibers was homogeneously dispersed in the above solution and ultrasonically treated for 1 h. Then, the mixture was placed into a stainless-steel autoclave and heated at 180 °C for 10 h. After the autoclave cooled to room temperature, the precipitate was recovered with the aid of a magnet and washed with deionized water and ethanol several times. Finally, the obtained C-nanofiber-coated magnetic nanoparticles were dried under vacuum at 70 °C for 6 h.

### 3.5. Samples Preparation

Fish, crab, and shrimp samples were collected from a local market and aquaculture base and determined to be free of the analytes before performing spiking and method validation studies. Fish were processed by discarding scales and skins, and then taking the muscle part along the back. Shrimp were processed by discarding the head, shell, and glands and keeping the muscle part. For crabs and shellfish, the edible part was taken and cut into pieces no larger than 0.5 cm × 0.5 cm × 0.5 cm. All samples were stored below −20 °C in a freezer after being homogenized in a high-speed food blender.

A total of 2.00 ± 0.01 g of homogenized samples were weighted into a 50 mL polypropylene centrifuge tube, and then 10 mL of glacial acetic acid–acetonitrile–water mixed extract (1:84:15, *v*/*v*) and 0.1 g Na_2_EDTA were added. The mixture was homogenized by vortex for 1 min and ultrasound for 15 min, followed by centrifuge at 8000 rpm for 5 min at 4 °C. The supernatant was collected, and the residue of the sample was re-extracted with 5 mL of glacial acetic acid–acetonitrile–water mixed extract (1:84:15, *v*/*v*). The liquid supernatant was collected and concentrated to nearly 2.5 mL using a stream of N_2_ at 40 °C and then diluted to 15 mL with water and filtered through a 0.45 μm membrane filter for the subsequent extraction procedure.

### 3.6. Magnetic Solid Phase Extraction

The synthesized carbon nanofiber magnetic materials were activated with 5.0 mL methanol and 5.0 mL water successively.

The MSPE procedure was performed as follows: 10 mg of activated carbon nanofiber magnetic material was directly added to the diluted supernatant, and the adsorption of TCs was carried out with parallel oscillation on a thermostat gas bath vibrator for 20 min. With the sorbent remaining in the tube, the supernatant was discarded with the help of the external magnet. Then, 4 mL eluate, which consisted of acetonitrile and 0.02 mol L^−1^ oxalic acid solution (1:8, *v*/*v*), was added into the tube to elute TCs from sorbents by parallel oscillating for 20 min. Subsequently, the eluate was separated from the sorbents with the help of the external magnet and was syringe-filtered into a little vial using a 0.22 μm nylon filter. A 10 μL aliquot was directly injected into the UHPLC-MS/MS system for analysis. The reusability of carbon nanofiber magnetic materials was evaluated by performing extraction and desorption repeatedly. The procedure for MSPE of TCs and subsequent analysis is shown in Figure 5b.

### 3.7. Method Validation

This analytical method was validated according to European Commission Decision 2002/657/EC [42]. Linearity, accuracy, precision, sensitivity, and the matrix effect of the proposed method were studied by analyzing standard fortified blank aquatic samples under optimized experiment conditions. The method validation was carried out using fish, crab, and shrimp samples previously examined and confirmed to be free of analytes. Three concentration levels (2.0 μg kg^−1^, 10.0 μg kg^−1^, and 50.0 μg kg^−1^) for each analyte were investigated for method validation.

Linearity was determined by analyzing the blank matrix spiked with serial concentrations of 4 kinds of TCs and plotting the peak areas versus concentrations. The recoveries were determined by spiking the samples with three different concentrations of the standard solutions and were calculated as measured content vs fortification level. Intra-day precision was determined by analyzing the spiked samples at three levels in five replicates on the same day, and inter-day precision was determined by running samples with spiked standards at the same levels over five separate days. The limit of detection (LOD) was determined based on 3 times the signal-to-noise ratio, while the limit of quantitation (LOQ) was obtained based on 10 times. The specificity of the method was investigated using 20 aquatic products with different kinds, which were confirmed to be free of the 4 kinds of TCs. These negative matrices were spiked with 4 kinds of TCs and analyzed with the proposed method, and the representative chromatograms of a standard solution, blank, and fortified fish sample were compared. The matrix effect was evaluated by analyzing five different aquatic products at 10 μg kg^−1^ concentration level for the 4 kinds of TCs and was measured by comparing the peak areas of analytes added post-extraction with pure standard solutions prepared in the mobile phase.

## 4. Conclusions

In the present work, Fe_3_O_4_@C–NF composites were synthesized with a one pot synthesis method and used as an adsorbent material for the high-efficiency MSPE of four kinds of TCs from aquatic products, followed by UHPLC-MS/MS determination. Characterization results suggested that Fe_3_O_4_@C–NFs had unique structures with good magnetic responsibility and large specific surface area, which endowed the material with tremendous capacity for the selective extraction of TCs in a complex matrix. MSPE conditions of the method were systemically studied to obtain the maximum extraction efficiency. With the assistance of UHPLC, a proper separation for the four kinds of TCs was achieved in less than 5 min. the method validation results showed that the developed method has relatively lower LODs, higher recoveries, and better precision and was convenient, economical, and may meet the requirements for trace TC determination in aquatic products. The established method expands the application of magnetic materials in the separation field of biosolid samples and has the potential to provide an alternative for the pretreatment of antibiotics in a complex matrix of animal-derived food samples.

## Figures and Tables

**Table 1:** Conditions of multiple reaction monitoring for the 4 TCs.

Analyte	Parent Ion (*m*/*z*)	Daughter Ion (*m*/*z*)	Cone Voltage (V)	Collision Energy (eV)
TC	445.1	410 *, 427	25	20, 14
OTC	461.1	426 *, 442.9	20	20, 15
CTC	479.1	443.9 *, 462	30	20, 18
DC	445.2	428 *, 154.1	30	18, 24

* quantitative ion.

**Table 2:** Linearity, sensitivity, and matrix effects of the proposed method and reproducibility of the Fe_3_O_4_@C–NF composites.

Analyte	Calibration Equation	Linear Range (ng/mL)	Correlation Efficient (R^2^)	LOD (μg/kg)	LOQ (μg/kg)	Reproducibility of the Composites (Inter-Batch Precision, RSD (%))
OTC	y = 6515.65x + 596.784	1.0~200	0.9991	0.7	2.0	5.71
TC	y = 7958.82x + 674.114	1.0~200	0.9992	0.7	2.0	6.02
CTC	y = 4449.41x − 533.554	1.0~200	0.9996	0.7	2.0	5.89
DC	y = 16990.5x + 5282.66	1.0~200	0.9997	0.7	2.0	6.32

**Table 3:** Results of recovery and precision of the spiked samples (*n* = 6).

Analyte	Spiked Level (μg/kg)	Grass Carp			Prawns			Sea Crab		
		Accuracy (%)	Intra-Day RSD (%)	Inter-Day RSD (%)	Accuracy (%)	Intra-Day RSD (%)	Inter-Day RSD (%)	Accuracy (%)	Intra-Day RSD (%)	Inter-Day RSD (%)
OTC	2.0	80.7	5.61	7.62	81.8	7.24	6.93	81.1	7.34	7.23
	10.0	82.5	4.73	5.33	89.0	6.51	4.74	85.7	6.61	9.04
	50.0	90.1	7.60	4.80	91.2	3.72	6.62	85.5	6.13	5.77
TC	2.0	81.3	6.28	7.88	88.7	7.33	4.82	86.6	4.72	7.13
	10.0	79.2	6.91	6.87	81.5	5.91	4.31	82.7	7.31	8.51
	50.0	84.6	5.54	8.41	81.7	6.77	8.84	92.8	6.24	5.42
CTC	2.0	87.1	4.81	6.51	91.9	7.71	9.68	98.2	5.82	6.48
	10.0	86.7	7.49	7.32	94.2	5.92	9.03	90.7	6.37	4.91
	50.0	90.2	6.62	6.71	90.4	6.31	7.51	97.7	8.01	4.20
DC	2.0	81.2	7.80	4.73	86.3	7.51	8.27	86.6	7.92	6.43
	10.0	83.1	8.17	7.22	90.7	5.50	4.72	87.3	6.33	6.81
	50.0	96.2	7.72	4.93	90.2	6.72	3.91	93.6	8.01	8.63

**Table 4:** Comparison of the proposed method with other reported methods for TCs analysis.

Extraction Method	Adsorbent/ Column Type	Sample/ Analytes	LOD	LOQ	Intra-Day RSD (%)	Inter-Day RSD (%)	Ref.
SPE/ HPLC–DAD	MIP	Eggs and pork /CTC,OTC,TC,DC	20–40 μg/kg	50–80 μg/kg	<8.1	/	[29]
SPE/HPLC-DAD	Strata-XL	Pig and other meat /CTC,OTC,TC,DC	5–10 μg/kg	25.0 μg/kg	<15.7	/	[30]
HPLC-FL	MgCl_2_	Fish muscle/ OTC,TC,CTC	/	1.0–1.5 μg/kg	<9.0	/	[31]
DLLME/ HPLC–DAD	MgSO_4_, NaCl	Veef/ OTC,TC,CTC	2.2–3.6 μg/kg	7.4–11.5 μg/kg	2–7	/	[32]
HPLC– DAD	/	Fish and shellfish/OTC,TC,CTC	15–62 μg/kg	125–175 μg/kg	<2	/	[33]
SPE/UHPLC– MS/MS	HLB	Swine muscle, swine liver, swine kidney, Chicken muscle, and bovine muscle/OTC,TC,CTC	0.5–4.0 μg/kg	2.0–10.0 μg/kg	<10	<14	[34]
SPE/HPLC-MS/MS	MIP	Lobster, duck, honey, and eggs/ OTC,TC,CTC,DC	0.1–0.3 μg/kg	0.2–1.1 μg/kg	<4.6	/	[35]
SPLE/HPLC-MS/MS	Copper(II) isonicotinate	Fatty-food samples/ OTC,TC,CTC,DC	0.2–3.3 μg/kg	/	5.5–13.6	/	[14]
HPLC-MS/MS	/	Fish/OTC,TC,CTC	10–16 μg/kg	33–52 μg/kg	<8	/	[36]
SPE/HPLC-MS/MS	Phospholipid	Fish/ OTC,TC	0.062–0.39 μg/kg	/	<20	/	[37]
MSPEDLLME/ HPLC–DAD	Fe_3_O_4_@ SiO_2_@ GO-β-CD	Water and milk/OTC,TC,DC	1.8 μg/L	6.1 μg/L	0.1–6.9	7.9	[26]
MSPE/HPLC	Fe_3_O_4_/ aptamer	Water and honey/TC	2.50 μg/L	10.0 μg/L	1.3	3.2	[38]
LLE-MSPE/ HPLC–UV	MNP-NH_2_	Milk/OTC,TC,CTC,DC	40 μg/L	50 μg/L	1.5	/	[39]
MSPE/HPLC– DAD	C-nanofiber-coated magnetic nanoparticles	Milk/TC	3.52 μg/L	9.83 μg/L	3.8	/	[27]
MSPE/HPLC– DAD	Cs-k-Fe_3_O_4_	Water/TC	0.21μg/L	0.63 μg/L	0.6–2.7	0.97–2.0	[25]
TDESs/HPLC– UV	TDESs	Water, honey, and milk/TC	1.0 μg/L	3.3 μg/L	2.8–4.1	3.6–5.2	[40]
MSPE/UHPLC– TUV	Fe_3_O_4_@SiO_2_@FeO	Water/TC	0.027–0.107 μg/L	/	0.1–1.9	1.0–3.8	[21]
MSPE/HPLC– DAD	rGO/Fe_3_O_4_	Water/TC	9.00 μg/L	29.0 μg/L	1.4	4.2	[41]
MSPE/UHPLC– MS/MS	Fe_3_O_4_@C–NFs	Aquatic products/ OTC,TC,CTC,DC	0.70 μg/kg	2.0 μg/kg	3.3–7.6	6.2–9.7	This work

## Data Availability

Data will be made available upon request.
